# Energy Intake from Healthy Foods Is Associated with Motor Fitness in Addition to Physical Activity: A Cross-Sectional Study of First-Grade Schoolchildren in Japan

**DOI:** 10.3390/ijerph19031819

**Published:** 2022-02-05

**Authors:** Naoko Hatta, Yuki Tada, Kazuko Ishikawa-Takata, Tadasu Furusho, Rieko Kanehara, Toshiki Hata, Azumi Hida, Yukari Kawano

**Affiliations:** 1 Graduate School of Agriculture, Tokyo University of Agriculture, Sakuragaoka 1-1-1, Setagaya, Tokyo 156-8502, Japan; nyako.705@gmail.com (N.H.); riekokk07@gmail.com (R.K.); 11320001@nodai.ac.jp (T.H.); 2 Department of Nutritional Science, Faculty of Applied Bioscience, Tokyo University of Agriculture, Sakuragaoka 1-1-1, Setagaya, Tokyo 156-8502, Japan; kt207460@nodai.ac.jp (K.I.-T.); a3hida@nodai.ac.jp (A.H.); y1kawano@nodai.ac.jp (Y.K.); 3 Department of International Food and Agricultural Science, Faculty of International Agriculture and Food Studies, Tokyo University of Agriculture, Sakuragaoka 1-1-1, Setagaya, Tokyo 156-8502, Japan; tfurusho@nodai.ac.jp

**Keywords:** children, motor fitness, diet, lifestyle habit

## Abstract

Childhood motor fitness is important for the physical and mental health of children and the prevention of future lifestyle diseases. This study aimed to investigate how energy intake from healthy foods and physical activity are associated with motor fitness among first-grade children. First-grade children (aged 6–7 years) attending three public elementary schools in Tokyo, Japan (*n* = 884), participated in this cross-sectional study. Self-administered questionnaires were distributed, which focused on lifestyle habits and required completion of a 1-day dietary record of meals that children ate at home. School lunch consumption was also assessed. Motor fitness was assessed by the New Physical Fitness Test (NPFT). Multiple regression analysis was used to investigate the association of the amount of energy from healthy foods and physical activity with NPFT scores. NPFT scores were positively correlated with involvement in after school exercise classes, playing outside (in boys only), and total energy intake. Energy intake from healthy foods showed a positively associated with NPFT scores (boys, β = 0.120, *p* = 0.011; girls, β = 0.140, *p* = 0.004), while energy intake from unhealthy foods did not. Energy intake from healthy foods was associated with motor fitness in children in addition to physical activity.

## 1. Introduction

Previous studies have found that cardiorespiratory endurance and muscular strength in childhood and adolescence are associated with systemic and abdominal fat, bone composition, and mental health [1]. García-Hermoso reviewed studies examining the association between muscular fitness during childhood and health parameters later in life and concluded that having high fitness reduced anthropometric and adiposity parameters, insulin resistance, and triglycerides [2]. Higher childhood motor fitness is important not only for the physical and mental health of children, but also for the prevention of future lifestyle diseases; thus, improving children’s motor fitness is vital.

The motor fitness of Japanese elementary school children had been growing until around 1985, but has been gradually declining since then, especially in their ability to run, jump and throw. The New Physical Fitness Test (NPFT) is implemented annually to Japanese primary school children, and the Ministry of Education, Culture, Sports, Science and Technology (MEXT) aims to exceed the standard set results of 1985, which indicated a record high for children’s motor fitness [3]. This reduction in motor fitness is concerning, as it could lead to a lack of exercise and reduced motor fitness in the future. 

Physical activity is one of the most important factors for achieving high motor fitness [4]. However, in addition to physical activity, the effect of dietary habits on motor fitness has been suggested [5]. One report indicates that adolescent children who eat breakfast every day have higher cardiorespiratory endurance than do children who skip breakfast [6]. Zaqout et al. [7] also demonstrated that consumption of fruit and vegetables is one of determinants of child’s motor fitness in addition to gender, body mass index (BMI), psychological factors, and amount of physical activity. Children in the growth phase must consume sufficient energy to support tissue synthesis and physical activity [8]. Previous studies have also reported that the content of food consumed by children is related to their physical and mental health [9,10]. However, most of these previous studies [6,7,10] reported on the consumption frequency of fruits and vegetables, or unhealthy foods, or evaluated the frequency of intake of limited food groups on a semi-quantitative basis, and did not assess whole dietary habit. 

We hypothesized that children who receive more energy from healthy food groups have a higher motor fitness level, as well as a higher physical activity level. To test this, we collected nationally standardized NPFT scores and dietary records from first-grade children in Japan. The present study aimed to investigate how healthy foods and physical activity are associated with motor fitness among first-grade children, after adjustment for several confounding factors.

## 2. Materials and Methods

A cross-sectional study of children and their guardians during the first year of compulsory education was conducted.

### 2.1. Participants

**Figure 1:**
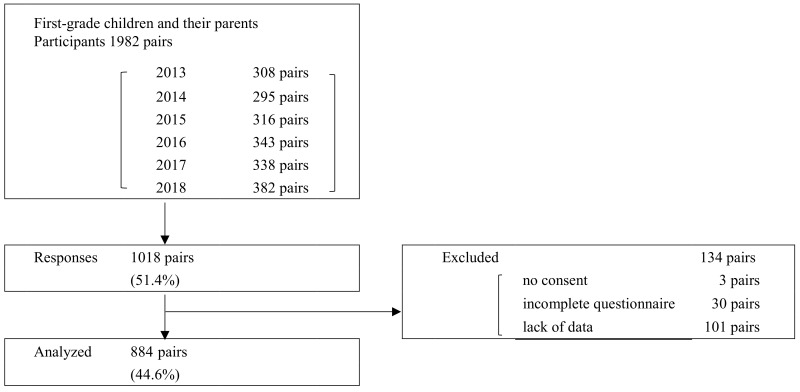
Flowchart of participant selection.

Participants were recruited from 1982 pairs of first-grade students aged 6–7 years in three public primary schools in Tokyo and their guardians between April 2013 and April 2018. Responses were obtained from 1018 pairs (51.4%). Of these, 884 pairs (51.2% boys and 48.8% girls) were analyzed after questionnaires with missing answers were excluded (Figure 1). This study was conducted according to the guidelines outlined in the Declaration of Helsinki. All procedures involving research study participants were approved by the Human Research Ethics Committees at Tokyo University of Agriculture (Reference numbers: 1122, 1130, 1501, 1605, 1704, 1802). Written informed consent was obtained from all participants.

### 2.2. Data Collection 

The researcher explained the aim and content of the survey to school principals and class teachers in each school. The class teachers distributed study protocols, consent forms, lifestyle questionnaires, and dietary records of meals that children ate at home. The survey forms were distributed annually between late May and early June, from 2013 to 2018, and were collected two weeks later. Consent forms and questionnaires were filled out at home, placed in an unsigned envelope, and submitted to each school. This study complied with the Strengthening the Reporting of Observational Studies in Epidemiology—nutrition epidemiology (STROBE-nut) guidelines [11].

### 2.3. Measures

#### 2.3.1. Motor Fitness (New Physical Fitness Test Score)

Motor fitness was assessed during the first term of every school year, based on the MEXT New Physical Fitness Test Implementation Guideline in each school [12]. The following items were measured: grip strength, sit-ups, sit and reach, side-steps, 20-metre shuttle run, 50-metre run, standing long jump, and softball throw (see Supplementary Materials). Elementary school teachers who are familiar with the measurement procedure conducted the test in their physical education classes, and the researchers received the data for the children participating in the study. Each measurement is allocated a score according to sex, in accordance with the Implementation Guideline. The total score for these eight items (NPFT score) was used as an index of motor fitness in the present study.

#### 2.3.2. Characteristics and Lifestyle Habits of Children 

Participating children and their guardians were instructed to complete a printed questionnaire on characteristics and lifestyle of children at home. Participating children were asked to indicate the frequency of playing outside at break time. Guardians were asked to provide information about the lifestyle of children: waking time; bedtime; frequency of eating breakfast per week; participation in after school classes including weekend classes; duration of watching television, video gaming, or reading comics, and studying per day at home. After school classes were categorised into the following three groups: exercise (e.g., baseball, soccer, swimming, gymnastics, basketball, tennis, or ballet), music (e.g., violin, piano, orchestra, drums, or chorus), and classroom learning [e.g., painting, science, language, juku (cram school), calligraphy, abacus, or religious studies]. Lesson time per week was calculated as the time spent at each session multiplied by the number of times each session is performed per week. Guardians were also asked to record their children’s height and weight measured at the health check-up conducted at each elementary school in April. A child’s physique was calculated using the following Rohrer’s index formula: weight (kg)/height (cm)^3^ × 10^7^.

#### 2.3.3. Children’s Dietary Habits

(1) Breakfast, dinner, and between-meal eating at home

Guardians filled out a dietary record to provide the information regarding the menu, ingredients, and weights of ingredients (including breakfast, dinner, and between-meal eating at home) for one average school day.

(2) Lunch (school lunch)

Japanese school lunches are provided every weekday based on school lunch implementation standards [13] in accordance with Dietary Reference Intakes for Japanese [8] set forth by the Ministry of Health, Labour and Welfare. Each school was surveyed for one day with regular menus. Investigators (registered dieticians and students in a nutrition course) observed children from the time they were served each dish up until the point they returned the trays. Consumption rate for each dish was evaluated using five categories (0% (food untouched), 25%, 50%, 75%, and 100% (completed)). Total intake was calculated based on the nutritional value of each menu and consumption rate for each child.

(3) Nutritional value calculation

Daily intake was calculated by combining intakes of breakfast, dinner, and between-meal eating at home with lunch intake. The energy content of surveyed meals was calculated using nutritional value calculation software (Excel Eiyo-kun, Version 8.0), with values based on the 2015 Standard Tables of Food Composition in Japan (Seventh Revised Version) [14]. Food consumed by children was divided into six food groups, according to the criteria in the Japanese Food Guide Spinning Top by the Ministry of Agriculture, Forestry and Fisheries and the Ministry of Health, Labour and Welfare [15], and numbers of servings were calculated. One serving (SV) was defined as the following: 40 g of carbohydrates for grain dishes; 70 g of the main ingredient in vegetable dishes; 6 g of protein in fish or meat dishes; 100 mg of calcium in milk or dairy products; 100 g of the main ingredient in fruit dishes; or 80 kcal of energy in confectionaries or sweetened beverages.

(4) Breakdown of energy intake

Energy intake from grain dishes, vegetable dishes, fish and meat dishes, milk and dairy products, and fruit was defined as energy from healthy foods. Energy intake from unhealthy foods was defined as the amount of energy derived from confectionaries and sweetened beverages. Confectionaries were classified according to the 2015 Standard Tables of Food Composition in Japan (Seventh Revised Version) [14]. Although the Standard Tables of Food Composition [14] classified ice cream as a dairy product, this study classified it as a confectionary. Similarly, this study categorized juice beverages that were not 100% fruit juice or that were from concentrate as sweetened beverages, in accordance with the Japanese Food Guide Spinning Top [15] definition of sweetened beverages.

#### 2.3.4. Statistical Analysis 

Participant characteristics were compared in relation to NPFT score quintile groups by gender with weighted one-way analyses of variance for continuous variables, and the Mantel–Haenszel test for trend for categorical variables.

A regression analysis adjusted for physique (Rohrer’s index) was conducted using the forced entry method for the investigation of items related to NPFT score quintile. When inputting dependant variables, total time spent watching television or participating in video gaming or reading comics was aggregated as sedentary activity at home, and classified into four categories (<60, 60–119, 120–179, ≥180 min/day). Study time (<15, 15–29, 30–44, 45–59, 60–74, 75–89, 90–104, ≥105 min/day), sleep duration (<8.0, 8.0–8.4, 8.5–8.9, 9.0–9.4, 9.5–9.9, 10.0–10.4, 10.5–10.9, ≥11 h/day) and Rohrer’s index were classified into eight nearly equal groups. Median values in each group were entered as dependent variables. The frequency of outside playing was set as ‘1’ for responses of “almost every day”; all other responses with a frequency less than “almost every day” were set as ‘0’. A value of ‘1’ was allocated for children engaged in after school exercise class, classroom learning, and/or music class, whereas children not engaged in after school classes including weekend classes were allocated dummy variables with a value of ‘0’. A value of ‘1’ was allocated for a response to the frequency of eating breakfast per week of “7 times a week”, whereas ‘0’ was allocated for all other responses. 

Moderator variables for investigation of the association between NPFT scores and daily energy intake and physical activity included the following: Rohrer’s index, sleep duration, time spent studying, sedentary activity at home, participation in after school classes (classroom learning or music), and breakfast eating. As for explanatory variables, in addition to playing outside and participation in after school exercise class, the amount of energy derived from healthy foods was used for model 1. The amount of energy derived from unhealthy foods was used for model 2, and the amounts of energy derived from both healthy foods and unhealthy foods were used for model 3. To demonstrate the appropriateness of using regression analysis, we verified the residuals of model 3 are normality distributed by using the Kolmogorov–Smirnov test. The G*power 3.1.9.7 program (Faul, F., Erdfelder, E., Buchner, A., & Lang, A.-G., Heinrich-Heine-Universität Düsseldorf, Germany), the power analysis software, determined the statistical power (1 − β) as >0.8 for the model 3. Data were expressed as means and standard deviations. A *p* value less than 0.05 was considered statistically significant. Statistical analysis was performed using IBM SPSS Statistics for Windows (Version 27.0).

## 3. Results

### 3.1. Characteristics of Participants

In this study, 453 (51.2%) of the participating children were boys and 431 (48.8%) were girls. Boys had higher values for height (*p* = 0.006) and weight (*p* = 0.001) than girls, but there was no significant difference in Rohrer’s index (*p* = 0.303). There was a sex difference in the results of all measurement items in the NPFT, except for sit-ups (*p* = 0.310), and boys had higher values in all items except for sit and reach (grip strength, *p* = 0.011; other items, *p* < 0.001). Overall NPFT scores were 30.0 ± 6.2 points for boys and 29.5 ± 6.2 points for girls, indicating no significant difference (*p* = 0.252).

Comparison of children’s characteristics and lifestyle habits in the five NPFT score quintiles indicated that boys with higher NPFT scores had higher heights (*p* < 0.001) and weights (*p* < 0.001). Boys with higher NPFT scores also had longer after school exercise lesson times (*p* < 0.001) and higher frequencies of playing outside (*p* < 0.001; Table 1). Girls with higher NPFT scores had longer after school exercise lesson times (*p* = 0.010) and lower Rohrer’s index values (*p* = 0.039; Table 2). 

### 3.2. Dietary Intake and New Physical Fitness Test Score

Boys with higher NPFT scores had higher total energy intake at lunch (*p* = 0.003), total daily energy intake (*p* = 0.013), energy intake derived from healthy foods (*p* = 0.003), vegetable dish intake (P = 0.001), and fish and meat dish intake (*p* = 0.028; Table 3). Girls with higher NPFT scores had higher values for total energy intake at breakfast (*p* = 0.008), energy intake from healthy foods at breakfast (*p* = 0.030), total energy intake at lunch (*p* < 0.001), total daily energy intake (*p* = 0.001), energy intake from healthy foods (*p* = 0.005), and fish and meat dish intake (*p* = 0.037; Table 4).

In boys, significant positive correlations were observed with playing outside [standardized partial regression coefficient (β) = 0.132, *p* = 0.005], after school exercise class (β = 0.139, *p* = 0.003), total daily energy intake (β = 0.151, *p* = 0.001), and energy intake from healthy foods (β = 0.170, *p* < 0.001; Table 5). In girls, significant positive correlations were observed with after school exercise class (β = 0.138, *p* = 0.004), total daily energy intake (β = 0.176, *p* < 0.001), and energy intake from healthy foods (β = 0.148, *p* = 0.002; Table 5). Multiple regression analysis was also conducted to assess the associations between NPFT scores and energy from healthy foods and unhealthy foods, and physical activity (Table 6). In boys, significant positive correlations were observed with energy intake from healthy foods (β = 0.151, *p* = 0.001; Model 1), in addition to playing outside (β = 0.104, *p* = 0.026) and after school exercise class (β = 0.126, *p* = 0.008). Energy intake from healthy foods was significantly correlated (β = 0.120, *p* = 0.011; Model 3) even after including unhealthy food intake. Similarly, in girls, significant positive correlations were observed with energy intake from healthy foods (β = 0.141, *p* = 0.004; Model 1), in addition to after school exercise class (β = 0.132, *p* = 0.006). Energy intake from healthy foods was also significantly correlated (β = 0.140, *p* = 0.004; Model 3) even after including for energy intake from unhealthy foods. Energy intake from unhealthy foods was not significantly correlated with NPFT scores in boys or girls.

## 4. Discussion

In the present study, NPFT scores were positively correlated with energy intake from healthy foods in children, in addition to physical activity. 

Energy intake from healthy foods had a significant positive correlation, and in particular, higher intake of vegetable dishes in boys and higher intake of fish and meat dishes in both boys and girls were correlated with NPFT scores. Previous studies have reported higher grip and lower limb muscle strength in groups with higher protein intake [16], and positive correlations between motor fitness and frequency of fruit and vegetable consumption [7]. The consumption of energy from grains, vegetables, and fish and meat, rather than from confectionaries and sweetened beverages, promotes healthier bodies and provides the required energy and nutrients in children.

No significant correlation was observed between energy derived from unhealthy foods and NPFT scores. The Japanese Food Guide Spinning Top defines an adequate intake of sweets and sweetened beverages as ≤200 kcal [15]. Means of energy intake from unhealthy foods for each of the five NPFT quintiles in the present study were all approximately 200 kcal. Although the quantitative effect of sweets and sweetened beverages is unknown, maintaining an appropriate level of intake for these food groups is important.

Participation in after school exercise class (an index of daily physical activity) was positively correlated with NPFT scores. Previous studies have reported that 13-year-old children who exercise three or more times per week have improved grip and 50-metre run [3], and that moderate-to-vigorous physical activity (associated with ≥3 metabolic equivalents) by school-age and adolescent children is positively correlated with motor fitness [7,17]. Exercise lesson programmes such as baseball, soccer, and swimming often include basic skill practice in addition to competition. Thus, children attending exercise classes regularly participate in activities such as side-stepping and sit-ups, as well as endurance enhancing exercises such as jogging. The strength developed during these activities is then reflected in activities included in physical fitness tests and may be positively correlated with NPFT scores. The present study also found a significant positive correlation between playing outside at break time at school or after school with NPFT scores in boys; a positive tendency was observed also in girls. At the time of the survey, the participating children were allowed to play outside freely based on their own intention at break time. Reports have shown that children with three or more days per week of outside playtime (another index of daily physical activity) participate in significantly longer periods of light and total physical activity, as compared to children who do not play outside as frequently [18]. Higher frequencies of playing outside, corresponding to larger amounts of physical activity, affected NPFT scores. 

Recent studies have shown that inadequate physical activity could be influenced by screen time use among children [19]. This study did not observe significant correlations between NPFT scores and sedentary activities at home, including the total time spent watching television and participating in video gaming or reading comics. According to a recent review, evidence is weak and inconsistent with respect to the association between screen time use and fitness [20]. Further studies will be needed to clarify the association between motor fitness and screen time use in children.

This study did not observe significant correlations between NPFT scores and other lifestyle factors that previously suggested. Sandercock et al. [6] reported higher cardiorespiratory endurance values in adolescent children who ate breakfast every day. However, only 67.9% of the study participants in the same study [6] ate breakfast every day, as compared to approximately 98% of participants in the present study. The percentage of children who missed breakfast one or more days per week in the current study was approximately 2%, which is a substantially smaller proportion than that observed previously. This difference may explain why the current study did not find breakfast consumption to be significantly correlated with NPFT scores. In the MEXT survey, children with eight or more hours of sleep per night had the highest NPFT scores [21]. However, previous studies that did not indicate correlations between sleep duration and motor fitness found mean sleep durations to be 9.6–9.7 h per night [7,17]. The present study found the mean sleep duration to be 9.6 h per night for both boys and girls, which supports the results of previous studies. Therefore, the lack of an observed correlation in this study may have been due to the children receiving adequate sleep. 

The strength of this study was that it investigated the correlations between motor fitness and nutritional intake among approximately 900 primary school students. The use of dietary records allowed for consideration of the mutual effects of meal quantity and quality. An additional strength was that motor fitness was assessed from the multiple components of fitness using the NPFT according to the MEXT guidelines. 

This study had several limitations. The present study was unable to include objective parameters of physical activities, such as actigraphy or pedometer use, due to the significant burden on participating teachers and children. Rather, the frequencies of playing outside and participating in after school exercise class were used. Because food intake was only surveyed for one day, the meals consumed on that day may not be indicative of children’s habitual meals. However, participating children and guardians were asked to enter data into the dietary record of meals that children ate at home that most closely represented meals on an average day, and school lunches were provided in accordance with Japanese school lunch implementation standards [13] and thus represent an average daily meal. In addition, data on the guardians’ educational backgrounds and household financial status could not be obtained. Finally, the response rate in this study was low at 44.6%. The survey may thus have had a selection bias, with a significant number of respondents who had a high degree of involvement in the survey and a high level of interest in participating children’s meals and health.

## 5. Conclusions

The present study found that energy from healthy foods is correlated with motor fitness in children in addition to their physical activity. This finding suggests that the consumption of healthy foods that include grains, vegetables, fruits, milk, and fish and meat, as well as exercise habits, are important for childhood motor fitness. 

## Figures and Tables

**Table 1:** Characteristics and lifestyle habits within New Physical Fitness Test score quintile groups among boys.

Variables	Median (Min-Max)	NPFT Score Quintile															*p* for Trend
		q1 (*n* = 88)			q2 (*n* = 67)			q3 (*n* = 93)			q4 (*n* = 106)			q5 (*n* = 99)			
		22.0			26.0			29.0			32.0			38.0			
		(13.0–24.0)			(25.0–27.0)			(28.0–30.0)			(31.0–34.0)			(35.0–49.0)			
Height	(cm)	116.3	±	5.7	117.8	±	5.5	117.6	±	5.1	118.8	±	4.7	119.3	±	4.8	<0.001
Weight	(m)	20.3	±	2.7	20.7	±	2.5	21.1	±	3.1	21.6	±	3.2	21.5	±	2.6	<0.001
Rohrer’s index	(kg/m^3^)	129.3	±	15.0	127.1	±	13.4	129.6	±	13.0	128.5	±	11.5	126.8	±	12.9	0.335
Grip strength	(kg)	6.7	±	1.9	7.8	±	1.8	8.2	±	1.7	8.7	±	1.9	9.8	±	2.1	<0.001
Sit-ups	(times)	7.0	±	4.8	9.8	±	4.6	11.5	±	4.5	12.7	±	3.1	15.6	±	4.6	<0.001
Sit and reach	(cm)	22.1	±	5.1	23.9	±	6.4	24.6	±	6.0	26.2	±	5.6	29.0	±	5.5	<0.001
Side-step	(points)	23.2	±	4.6	24.1	±	5.4	26.1	±	4.7	28.2	±	4.1	31.6	±	3.9	<0.001
20-metre shuttle run	(times)	11.9	±	4.2	17.2	±	7.7	17.7	±	7.1	22.6	±	11.1	28.6	±	13.8	<0.001
50-metre run	(s)	12.3	±	0.9	11.8	±	0.7	11.5	±	0.6	10.9	±	0.6	10.6	±	0.5	<0.001
Standing long jump	(cm)	97.5	±	13.5	104.4	±	12.8	110.0	±	13.7	115.3	±	11.3	127.3	±	11.7	<0.001
Softball throw	(m)	5.8	±	2.2	6.1	±	2.1	7.7	±	2.5	7.9	±	2.7	10.5	±	3.5	<0.001
Sleep duration	(hours/day)	9.5	±	0.6	9.6	±	0.7	9.5	±	0.6	9.8	±	0.7	9.6	±	0.7	0.278
Studying	(min/day)	49.1	±	23.7	44.3	±	19.1	42.7	±	21.3	47.4	±	44.7	44.6	±	21.8	0.529
Watching television	(min/day)	78.5	±	61.3	74.5	±	46.4	83.8	±	46.2	70.2	±	41.0	75.3	±	42.9	0.442
Playing video games/reading comics	(min/day)	20.2	±	25.3	23.2	±	40.9	28.1	±	31.0	17.2	±	24.1	23.3	±	23.2	0.973
After school exercise class	(min/week)	81.8	±	118.2	104.2	±	107.7	114.1	±	126.6	155.7	±	143.9	195.7	±	167.9	<0.001
After school classroom learning	(min/week)	62.7	±	72.5	61.3	±	79.8	45.3	±	80.1	47.7	±	65.9	47.9	±	59.7	0.079
After school music class	(min/week)	9.0	±	24.2	9.6	±	21.8	8.6	±	20.8	5.9	±	21.0	6.8	±	17.4	0.258
Playing outside	Always	74	(84.1)		61	(91.0)		87	(93.5)		102	(96.2)		95	(96.0)		<0.001
Frequency of eating breakfast	0–2 times/week	0	(0.0)		0	(0.0)		0	(0.0)		0	(0.0)		0	(0.0)		0.866
	3–4 times/week	0	(0.0)		0	(0.0)		0	(0.0)		0	(0.0)		1	(1.0)		
	5–6 times/week	2	(2.3)		1	(1.5)		2	(2.2)		1	(0.9)		1	(1.0)		
	7 times/week	86	(97.7)		66	(98.5)		91	(97.8)		105	(99.1)		97	(98.0)		

Mean ± SD or number (%), *p* values are based on weighted one-way analysis of variance for continuous variables, or the Mantel-Haenszel test for trend for categorical variables. NPFT, New Physical Fitness Test.

**Table 2:** Characteristics and lifestyle habits within New Physical Fitness Test score quintile groups among girls.

Variables		NPFT Score Quintile															*p* for Trend
		q1 (*n* = 70)			q2 (*n* = 96)			q3 (*n* = 82)			q4 (*n* = 91)			q5 (*n* = 92)			
	Median (Min-Max)	21.0			26.0			29.0			33.0			38.0			
		(9.0–23.0)			(24.0–27.0)			(28.0–30.0)			(31.0–34.0)			(35.0–46.0)			
Height	(cm)	114.2	±	5.5	115.1	±	5.4	117.5	±	4.6	117.7	±	4.4	118.5	±	4.7	<0.001
Weight	(m)	19.5	±	2.9	20.0	±	3.0	20.9	±	3.1	20.9	±	2.9	21.3	±	2.9	<0.001
Rohrer’s index	(kg/m^3^)	131.0	±	13.8	131.2	±	14.5	128.2	±	13.4	127.8	±	12.1	128.0	±	13.0	0.039
Grip strength	(kg)	6.3	±	2.1	7.4	±	1.6	7.7	±	1.5	8.4	±	2.0	9.6	±	2.1	<0.001
Sit-ups	(times)	6.5	±	5.1	10.0	±	5.1	11.1	±	4.2	12.4	±	4.2	14.9	±	3.6	<0.001
Sit and reach	(cm)	22.9	±	6.4	27.0	±	6.2	28.5	±	5.3	30.0	±	6.5	32.8	±	6.5	<0.001
Side-step	(points)	22.2	±	6.5	24.0	±	4.8	25.7	±	3.9	27.1	±	3.3	28.3	±	3.9	<0.001
20-metre shuttle run	(times)	11.0	±	5.2	14.4	±	6.6	15.6	±	6.7	18.7	±	8.1	23.4	±	9.4	<0.001
50-metre run	(s)	12.7	±	1.9	12.1	±	0.7	11.6	±	0.6	11.3	±	0.7	10.8	±	0.5	<0.001
Standing long jump	(cm)	90.7	±	20.9	99.5	±	13.3	103.8	±	11.5	108.2	±	12.6	118.4	±	11.5	<0.001
Softball throw	(m)	3.9	±	1.5	4.4	±	1.5	5.2	±	1.6	5.8	±	1.6	6.4	±	1.9	<0.001
Sleep duration	(hours/day)	9.5	±	0.7	9.7	±	0.6	9.5	±	0.6	9.6	±	0.5	9.6	±	0.7	0.666
Studying	(min/day)	41.9	±	19.9	48.9	±	25.0	46.9	±	34.3	41.2	±	17.4	44.8	±	32.7	0.679
Watching television	(min/day)	64.3	±	41.6	74.2	±	45.0	72.5	±	48.2	66.8	±	42.9	66.0	±	49.2	0.654
Playing video games/reading comics	(min/day)	15.0	±	25.4	14.8	±	27.5	15.3	±	28.7	12.9	±	20.8	12.4	±	23.2	0.397
After school exercise class	(min/week)	54.3	±	61.1	73.4	±	84.6	93.9	±	106.3	82.3	±	90.1	96.1	±	131.4	0.010
After school classroom learning	(min/week)	47.0	±	78.8	59.3	±	77.6	61.2	±	66.4	56.7	±	91.1	39.5	±	60.1	0.379
After school music class	(min/week)	16.1	±	30.2	22.5	±	38.6	24.8	±	32.7	27.9	±	36.5	23.2	±	35.9	0.145
Playing outside	Always	60	(85.7)		83	(86.5)		73	(89.0)		86	(94.5)		84	(91.3)		0.069
Frequency of eating breakfast	0–2 times/week	0	(0.0)		0	(0.0)		0	(0.0)		0	(0.0)		0	(0.0)		0.397
	3–4 times/week	1	(1.4)		1	(1.0)		1	(1.2)		0	(0)		0	(0.0)		
	5–6 times/week	0	(0.0)		3	(3.1)		3	(3.7)		3	(3.3)		1	(1.1)		
	7 times/week	69	(98.6)		92	(95.8)		78	(95.1)		88	(96.7)		91	(98.9)		

Mean ± SD or number (%), *p* values are based on weighted one-way analysis of variance for continuous variables, or the Mantel-Haenszel test for trend for categorical variables. NPFT, New Physical Fitness Test.

**Table 3:** Dietary intake within New Physical Fitness Test score quintile groups among boys.

Variables		NPFT Score Quintile															*p* for Trend
		q1 (*n* = 88)			q2 (*n* = 67)			q3 (*n* = 93)			q4 (*n* = 106)			q5 (*n* = 99)			
Breakfast																	
Total energy	(kcal)	355	±	114	383	±	123	362	±	129	377	±	146	374	±	122	0.387
Energy from healthy foods *	(kcal)	321	±	125	352	±	133	323	±	122	334	±	138	332	±	134	0.819
Energy from unhealthy foods	(kcal)	35	±	81	31	±	61	39	±	81	43	±	97	42	±	107	0.362
Lunch																	
Total energy	(kcal)	478	±	137	476	±	117	490	±	135	516	±	137	531	±	134	0.003
Dinner																	
Total energy	(kcal)	538	±	169	627	±	214	562	±	195	560	±	178	609	±	178	0.152
Energy from healthy foods *	(kcal)	530	±	168	611	±	205	551	±	194	552	±	176	597	±	177	0.153
Energy from unhealthy foods	(kcal)	7	±	11	17	±	34	11	±	18	8	±	14	12	±	19	0.815
Between meal eating																	
Total energy	(kcal)	208	±	159	178	±	129	213	±	165	210	±	143	194	±	152	0.951
Energy from healthy foods *	(kcal)	52	±	96	40	±	70	41	±	80	70	±	93	66	±	125	0.071
Energy from unhealthy foods	(kcal)	156	±	138	138	±	121	172	±	132	140	±	128	128	±	117	0.151
Daily																	
Total energy	(kcal)	1579	±	321	1665	±	306	1628	±	369	1663	±	307	1708	±	315	0.013
Energy from healthy foods *	(kcal)	1381	±	274	1479	±	282	1406	±	319	1472	±	294	1526	±	319	0.003
Energy from unhealthy foods	(kcal)	198	±	177	185	±	140	222	±	161	191	±	156	182	±	170	0.548
Grain dishes	(SV)	2.90	±	0.85	2.87	±	0.76	2.83	±	0.76	2.99	±	0.85	3.11	±	1.06	0.055
Vegetable dishes	(SV)	3.39	±	1.38	3.89	±	1.62	3.62	±	1.70	4.12	±	2.08	4.25	±	1.91	0.001
Fish and meat dishes	(SV)	4.42	±	1.79	5.16	±	2.03	4.66	±	1.86	4.89	±	2.01	5.22	±	2.11	0.028
Milk and dairy products	(SV)	3.67	±	1.41	4.06	±	2.23	3.72	±	1.92	3.96	±	1.81	3.84	±	2.06	0.643
Fruits	(SV)	0.70	±	0.78	0.86	±	0.92	0.78	±	0.85	0.87	±	0.98	0.78	±	0.71	0.485
Confectionaries and sweetened beverages	(SV)	2.58	±	2.24	2.44	±	1.75	2.87	±	2.01	2.50	±	1.94	2.39	±	2.12	0.554

Mean ± SD, *p* values are based on weighted one-way analysis of variance. NPFT, New Physical Fitness Test. * Energy intake derived from grain dishes, vegetable dishes, fish and meat dishes, milk and dairy products, and fruit (based on the food groups defined by the Japanese Food Guide Spinning Top), as opposed to confectionaries and sweetened beverages.

**Table 4:** Dietary intake within New Physical Fitness Test score quintile groups among girls.

Variables		NPFT Score Quintile															*p* for Trend
		q1 (*n* = 70)			q2 (*n* = 96)			q3 (*n* = 82)			q4 (*n* = 91)			q5 (*n* = 92)			
Breakfast																	
Total energy	(kcal)	317	±	119	323	±	125	328	±	107	353	±	124	355	±	105	0.008
Energy from healthy foods *	(kcal)	289	±	133	286	±	123	294	±	125	333	±	129	311	±	118	0.030
Energy from unhealthy foods	(kcal)	28	±	63	37	±	75	34	±	67	20	±	41	44	±	91	0.606
Lunch																	
Total energy	(kcal)	417	±	126	480	±	127	448	±	110	470	±	115	520	±	138	<0.001
Dinner																	
Total energy	(kcal)	538	±	148	524	±	158	502	±	163	536	±	221	535	±	172	0.848
Energy from healthy foods *	(kcal)	527	±	144	515	±	155	492	±	156	524	±	219	524	±	170	0.875
Energy from unhealthy foods	(kcal)	10	±	18	10	±	13	10	±	18	13	±	25	10	±	13	0.515
Between meal eating																	
Total energy	(kcal)	166	±	126	190	±	148	178	±	157	194	±	137	173	±	114	0.780
Energy from healthy foods *	(kcal)	37	±	65	52	±	98	52	±	109	44	±	74	31	±	62	0.375
Energy from unhealthy foods	(kcal)	129	±	110	138	±	124	126	±	125	150	±	123	142	±	104	0.338
Daily																	
Total energy	(kcal)	1438	±	270	1517	±	278	1457	±	278	1554	±	329	1583	±	262	0.001
Energy from healthy foods *	(kcal)	1271	±	265	1333	±	275	1287	±	264	1372	±	310	1387	±	251	0.005
Energy from unhealthy foods	(kcal)	167	±	127	184	±	140	170	±	142	184	±	142	196	±	149	0.254
Grain dishes	(SV)	2.65	±	0.78	2.72	±	0.77	2.69	±	0.75	2.72	±	0.75	2.86	±	0.79	0.111
Vegetable dishes	(SV)	3.63	±	1.48	3.92	±	1.59	3.61	±	1.63	3.85	±	1.60	4.13	±	1.65	0.101
Fish and meat dishes	(SV)	4.06	±	1.52	4.28	±	1.65	4.37	±	2.11	4.66	±	1.99	4.54	±	1.69	0.037
Milk and dairy products	(SV)	3.34	±	1.52	3.53	±	1.67	2.89	±	1.51	3.61	±	1.64	3.37	±	1.60	0.805
Fruits	(SV)	0.74	±	0.79	0.61	±	0.79	0.77	±	0.80	0.87	±	0.93	0.70	±	0.89	0.516
Confectionaries and sweetened beverages	(SV)	2.17	±	1.59	2.40	±	1.74	2.21	±	1.76	2.39	±	1.77	2.56	±	1.85	0.207

Mean ± SD, *p* values are based on weighted one-way analysis of variance. NPFT, New Physical Fitness Test. * Energy intake derived from grain dishes, vegetable dishes, fish and meat dishes, milk and dairy products, and fruit (based on the food groups defined by the Japanese Food Guide Spinning Top), as opposed to confectionaries and sweetened beverages.

**Table 5:** Linear regression analysis to investigate factors related to New Physical Fitness Test score.

Variables	Boys		Girls	
	β *	*p*	β *	*p*
Sleep duration	0.019	0.684	0.002	0.967
Playing outside	0.132	0.005	0.082	0.088
Studying at home	−0.059	0.210	−0.021	0.667
Sedentary activity at home	−0.007	0.877	−0.006	0.895
After school exercise class	0.139	0.003	0.138	0.004
After school classroom learning	0.008	0.860	0.011	0.823
After school music class	−0.013	0.775	0.079	0.104
Eating breakfast every day	−0.013	0.774	0.003	0.944
Daily total energy intake	0.151	0.001	0.176	<0.001
Energy intake from healthy foods ^†^	0.170	<0.001	0.148	0.002
Energy intake from unhealthy foods	−0.015	0.751	0.071	0.138

* Standardized regression coefficient adjusted for Rohrer’s index. Sleep duration was classified into eight categories. The frequency of playtime outside was set as ‘1’ for responses of “almost every day”; all other responses were set as dummy variables with a value of ‘0’. Study time was classified into eight categories. Sedentary activity at home included total time spent watching television and participating in video gaming or reading comics, and was classified into four categories. A value of ‘1’ was allocated for children engaged in after school exercise class, classroom learning, and/or music class, whereas children not engaged in after school classes including weekend classes were allocated dummy variables with a value of ‘0’. A value of ‘1’ was allocated for eating breakfast every day, whereas other responses were allocated dummy variables with a value of ‘0’. ^†^ Energy intake derived from grain dishes, vegetable dishes, fish and meat dishes, milk and dairy products, and fruits (based on the food groups defined by the Japanese Food Guide Spinning Top), as opposed to confectionaries and sweetened beverages.

**Table 6:** Multiple regression analysis for New Physical Fitness Test score.

Variables	Model 1		Model 2		Model 3	
	β ^†^	*p*	β ^†^	*p*	β ^†^	*p*
Boys						
Playing outside	0.104	0.026	0.122	0.010	0.131	0.005
After school exercise class	0.126	0.008	0.137	0.004	0.133	0.005
Energy from healthy foods *	0.151	0.001	-	-	0.120	0.011
Energy from unhealthy foods	-	-	−0.022	0.638	−0.019	0.683
Girls						
Playing outside	0.082	0.088	0.088	0.070	0.088	0.066
After school exercise class	0.132	0.006	0.139	0.004	0.120	0.013
Energy from healthy foods *	0.141	0.004	-	-	0.140	0.004
Energy from unhealthy foods	-	-	0.063	0.195	0.076	0.116

Each model was adjusted for Rohrer’s index, sleep duration, study time, the sedentary activities at home, participation in after school classes (classroom learning and/or music), and eating breakfast every day. ^†^ Standardized regression coefficient. * Energy intake derived from grain dishes, vegetable dishes, fish and meat dishes, milk and dairy products, and fruits (based on the food groups defined by the Japanese Food Guide Spinning Top), as opposed to confectionaries and sweetened beverages.

## Data Availability

The datasets used and/or analysed during the current study are available from the corresponding author on reasonable request.

## Supplementary Materials

The following supporting information can be downloaded at: https://www.mdpi.com/article/10.3390/ijerph19031819/s1, Brief summary of the New Physical Fitness Test Implementation Guideline.
