# Supplementary material for: Energy Intake from Healthy Foods Is Associated with Motor Fitness in Addition to Physical Activity: A Cross-Sectional Study of First-Grade Schoolchildren in Japan
Source: Int J Environ Res Public Health. 2022 Feb 5;19(3):1819. doi: 10.3390/ijerph19031819 (PMC8834963; doi:10.3390/ijerph19031819)
Supplement: Supplementary file 1 [file ijerph-19-01819-s001.zip › ijerph-1566711-supplementary.pdf]

Supplementary
Brief summary of the New Physical Fitness Test Implementation Guideline

(1) Grip strength

Grip strength measurements were performed using a Smedley-type hand dynamometer. Children were positioned standing upright with feet placed a natural distance apart and arms by the sides of the body. They were then instructed to grip the hand dynamometer with all their strength to prevent the dynamometer from touching their body. The hand dynamometer was held tight to prevent it from swaying and the exercise was repeated twice on both the left and right sides. Scores were calculated as averages of the better results from each side. Boys were allocated the following point values: ≤4 kg = 1 point, 5–6 kg = 2 points, 7–8 kg = 3 points, 9–10 kg = 4 points, 11–13 kg = 5 points, 14–16 kg = 6 points, 17–19 kg = 7 points, 20–22 kg = 8 points, 23–25 kg = 9 points, and ≥26 kg = 10 points(16). Girls were allocated the following point values: ≤3 kg = 1 point, 4–6 kg = 2 points, 7–8 kg = 3 points, 9–10 kg = 4 points, 11–12 kg = 5 points, 13–15 kg = 6 points, 16–18 kg = 7 points, 19–21 kg = 8 points, 22–24 kg = 9 points, and ≥25 kg = 10 points.

(2) Sit-ups

Sit-up exercises were performed with children lying on their backs, gripping both hands lightly with arms crossed in front of their chests, and knees bent at a 90° angle. An assistant was assigned to hold the children’s knees. When given the signal to start, children were instructed to lift the upper body from a reclined position to an upright position until both elbows touched both thighs, and then to return to the starting position. This action was repeated as many times as

possible in 30 s. This test was implemented once and the number of times the action was repeated within 30 s was recorded. Boys were allocated the following point values: ≤2 times = 1 point, 3–5 times = 2 points, 6–8 times = 3 points, 9–11 times = 4 points, 12–14 times = 5 points, 15–17 times = 6 points, 18–19 times = 7 points, 20–22 times = 8 points, 23–25 times = 9 points, and ≥ 26 times = 10 points(16). Girls were allocated the following point values: ≤2 times = 1 point, 3–5 times = 2 points, 6–8 times = 3 points, 9–11 times = 4 points, 12–13 times = 5 points, 14–15 times = 6 points, 16–17 times = 7 points, 18–19 times = 8 points, 20–22 times = 9 points, and ≥23 times = 10 points .

(3) Sit and reach

Sit and reach measurements were performed using a stand made of two boxes approximately 25 cm in height that were joined across by a piece of cardboard across the top of both boxes. Children sat with both legs straight in front of them, between the two boxes, with their back and buttocks flush against a wall. They were instructed to place both hands flat on the nearest side of the cardboard, and to expand the chest with elbows straight to lengthen the spine. Children then bent forward from the starting position, without removing their hands, in order to slide the box as far forward as they could reach. The distance that the box was moved from the starting position was then measured with a ruler. This exercise was repeated twice, and the best result was recorded. Boys were allocated the following point values: ≤14 cm = 1 point, 15–18 cm = 2 points, 19–22 cm = 3 points, 23–26 cm = 4 points, 27–29 cm = 5 points, 30–33 cm = 6 points, 34–37 cm = 7 points, 38–42 cm = 8 points, 43–48 cm = 9 points, and ≥ 49 cm = 10 points(16). Girls were allocated the

following point values: ≤17 cm = 1 point, 18–20 cm = 2 points, 21–24 cm = 3 points, 25–28 cm = 4 points, 29–32 cm = 5 points, 33–36 cm = 6 points, 37–40 cm = 7 points, 41–45 cm = 8 points, 46–51 cm = 9 points, and ≥52 cm = 10 points .

(4) Side-steps

A central line was drawn on the floor with two parallel lines drawn 100 cm to either side of the central line. Children were instructed to stand with their feet on either side of the central line and, when given the signal to start, to step sideways to straddle the right line, return to straddle the central line, and similarly step sideways to straddle the left line. The exercise was continued until children stepped either outside of or on the lines. Children repeated this exercise as many times as possible for 20 s, and the number of times any line was crossed was recorded. This test was repeated twice, and the best result was recorded. Boys were allocated the following point values: ≤17 times = 1 point, 18–21 times = 2 points, 22–25 times = 3 points, 26–29 times = 4 points, 30–33 times = 5 points, 34–37 times = 6 points, 38–41 times = 7 points, 42–45 times = 8 points, 46–49 times = 9 points, and ≥50 times = 10 points(16). Girls were allocated the following point values: ≤16 times = 1 point, 17–20 times = 2 points, 21–24 times = 3 points, 25–27 times = 4 points, 28–31 times = 5 points, 32–35 times = 6 points, 36–39 times = 7 points, 40–42 times = 8 points, 43–46 times = 9 points, and ≥47 times = 10 points .

(5) 20-m shuttle run

Children were instructed to run back and forth between two parallel lines placed 20 m apart in time with auditory prompts from a CD player. When prompts were played at fixed intervals, the children ran 20 m to the opposite line. When their feet touched or went past the line, they turned around to await the next prompt, and repeated the exercise. The intervals between auditory prompts were shortened approximately every minute, and children attempted to reach the opposite line before hearing the next prompt. The number of times that each child was able to run 20 m in time with the prompts was recorded as the total number of complete times. This exercise was performed once. Boys were allocated the following point values: ≤7 times = 1 point, 8–9 times = 2 points, 10–14 times = 3 points, 15–22 times = 4 points, 23–32 times = 5 points, 33–44 times = 6 points, 45–56 times = 7 points, 57–68 times = 8 points, 69–79 times = 9 points, and ≥80 times = 10 points. Girls were allocated the following points: ≤7 times = 1 point, 8–9 times = 2 points, 10–13 times = 3 points, 14–18 times = 4 points, 19–25 times = 5 points, 26–34 times = 6 points, 35–43 times = 7 points, 44–53 times = 8 points, 54–63 times = 9 points, and ≥64 times = 10 points.

(6) 50-m run

Children were instructed to run 50 m from a standing start. Times were recorded in 0.1-s units, with less than 0.1-s units rounded up. This exercise was performed once. Boys were allocated the following point values: ≥13.1 s = 1 point, 12.3–13.0 s = 2 points, 11.5–12.2 s = 3 points, 10.7–11.4 s = 4 points, 10.0–10.6 s = 5 points, 9.4–9.9 s = 6 points, 8.9–9.3 s = 7 points, 8.5–8.8 s = 8

points, 8.1–8.4 s = 9 points, and ≤8.0 s = 10 points. Girls were allocated the following point values: ≥13.3 s = 1 point, 12.5–13.2 s = 2 points, 11.7–12.4 s = 3 points, 11.0–11.6 s = 4 points, 10.3–10.9 s = 5 points, 9.7–10.2 s = 6 points, 9.2–9.6 s = 7 points, 8.8–9.1 s = 8 points, 8.4–8.7 s = 9 points, and ≤8.3 s = 10 points.

(7) Standing long jump

Children were instructed to stand with feet slightly apart, and the tips of their toes aligned with the front edge of the take-off line. They then jumped with both feet in unison onto a sandpit or mat in front of the line. The landing position was measured based on the point closest to the take-off line and the distance between that point and the central point between both feet on the take-off line is measured in a straight line. This test was repeated twice, and the best result was recorded. Boys were allocated the following point values: ≤92 cm = 1 point, 93–104 cm = 2 points, 105–116 cm = 3 points, 117–129 cm = 4 points, 130–142 cm = 5 points, 143–155 cm = 6 points, 156–167 cm = 7 points, 168–179 cm = 8 points, 180–191 cm = 9 points, and ≥192 cm = 10 points . Girls were allocated the following point values: ≤84 cm = 1 point, 85–97 cm = 2 points, 98–108 cm = 3 points, 109–120 cm = 4 points, 121–133 cm = 5 points, 134–146 cm = 6 points, 147–159 cm = 7 points, 160–169 cm = 8 points, 170–180 cm = 9 points, and ≥181 cm = 10 points .

(8) Softball throw

Children were instructed to throw a number one size softball (measuring 26.7 ± 0.32 cm in circumference). The distance to the location at which the ball landed within pre-drawn arcs at one-

metre intervals was measured. This test was repeated twice, and the best result was recorded. Boys were allocated the following point values: ≤4 m = 1 point, 5–6 m = 2 points, 7–9 m = 3 points, 10–12 m = 4 points, 13–17 m = 5 points, 18–23 m = 6 points, 24–29 m = 7 points, 30–34 m = 8 points, 35–39 m = 9 points, and ≥40 m = 10 points[16]. Girls were allocated the following point values: ≤3 m = 1 point, 4 m = 2 points, 5 m = 3 points, 6–7 m = 4 points, 8–10 m = 5 points, 11–13 m = 6 points, 14–16 m = 7 points, 17–20 m = 8 points, 21–24 m = 9 points, and ≥25 m = 10 points.
